# Lymphatic system regulation of anti-cancer immunity and metastasis

**DOI:** 10.3389/fimmu.2024.1449291

**Published:** 2024-08-15

**Authors:** Pin-Ji Lei, Cameron Fraser, Dennis Jones, Jessalyn M. Ubellacker, Timothy P. Padera

**Affiliations:** ^1^ Edwin L. Steele Laboratories for Tumor Biology, Department of Radiation Oncology, Massachusetts General Hospital Cancer Center, Massachusetts General Hospital and Harvard Medical School, Boston, MA, United States; ^2^ Department of Molecular Metabolism, Harvard T.H. Chan School of Public Health, Boston, MA, United States; ^3^ Department of Pathology and Laboratory Medicine, Boston University School of Medicine, Boston, MA, United States

**Keywords:** lymphatic vessels, lymph node metastasis, tumor-draining lymph node (TDLN), immunotherapy, cancer metastasis

## Abstract

Cancer dissemination to lymph nodes (LN) is associated with a worse prognosis, increased incidence of distant metastases and reduced response to therapy. The LN microenvironment puts selective pressure on cancer cells, creating cells that can survive in LN as well as providing survival advantages for distant metastatic spread. Additionally, the presence of cancer cells leads to an immunosuppressive LN microenvironment, favoring the evasion of anti-cancer immune surveillance. However, recent studies have also characterized previously unrecognized roles for tumor-draining lymph nodes (TDLNs) in cancer immunotherapy response, including acting as a reservoir for pre-exhausted CD8+ T cells and stem-like CD8+ T cells. In this review, we will discuss the spread of cancer cells through the lymphatic system, the roles of TDLNs in metastasis and anti-cancer immune responses, and the therapeutic opportunities and challenges in targeting LN metastasis.

## Introduction

Cancer cell invasion of lymphatic vessels (1, 2) and the presence of metastatic cancer cells in sentinel LNs indicate that cancer has progressed to an advanced stage, which can guide treatment options and impact the overall prognosis (3–7). A higher number of affected LNs is associated with an increased risk of cancer recurrence and a worsened survival rate in many cancer types, including melanoma (8, 9), breast cancer (10, 11), head and neck cancer (12), prostate cancer (13, 14), and colon cancer (15, 16). Due to the significant importance of LN status in cancer staging, sentinel LN biopsy (SLNB) is generally recommended as standard staging practice in various solid tumors (4, 17–21). Further, in patients with clinically evident disease in lymph nodes, complete LN dissection (CLND) is often recommended to remove potentially involved LNs. These clinical decisions assume that surgical removal of metastatic LNs could inhibit the further spreading of cancer cells. However, findings from several recent clinical trials have challenged this hypothesis (22–28). These studies indicate that CLND does not significantly improve the overall survival rates in melanoma and breast cancer patients with early-stage disease and positive SLNB. The lymphadenectomy procedures can lead to severe post-operative complications, including infections and lymphedema (29). Thus, the decision to surgically remove LNs warrants careful consideration. Clinical decision-making has become further complicated by the emerging significance of TDLNs for tumor antigen presentation (30), T cell priming (31–33), immunotherapy response (34–36), cancer vaccines (37–39) and CAR-T therapy (40–42), underscoring the need to consider the implications of LN removal on immunotherapy.

Lymphatic transport of antigen-presenting cells (APCs) and soluble tumor antigens to TDLNs is critical for the initiation of an anti-cancer immune response (43). The TDLNs are the first site where dendritic cells present tumor-antigens to CD8+ T cells to prime and activate them (44–46). The activated CD8+ T cells are subsequently recruited to the primary tumor sites. However, most intratumoral CD8+ T cells become exhausted rapidly due to the immunosuppressive tumor microenvironment (47). The circulation of TCF-1+ stem-like CD8+ T cells between the primary tumor and TDLNs is critical for systemic and intratumoral immune responses (47). Notably, the presence of stem-like CD8+ T cells in the TDLNs has been associated with durable treatment responses (31). Moreover, anti-PD-1 monoclonal antibodies not only act at the primary tumor site but also display additional activity in the periphery (48–50), particularly in the TDLNs (30, 51, 52), promoting the magnitude and quality of tumor antigen-specific CD8+ T cell responses in TDLNs (33).

To evade the important role of TDLNs in anti-cancer immunity, the progression and metastasis of cancer cells to LNs cause an immunosuppressive LN microenvironment (53–59). The elevation of Type I and Type II interferon-signaling pathways in cancer cells promotes their survival by evading NK cell killing (57) and suppressing effector T cell function by enhancing the accumulation of T regulatory (Treg) cells in the LNs (59). Moreover, the LN microenvironment accelerates epigenetic (60) and metabolic reprogramming of cancer cells (61, 62) that promote resistance to ferroptosis, enhancing survival and metastasis through the blood (62). Understanding how cancer cells escape immune surveillance in the LN, and how interactions between cancer cells and the LN microenvironment lead to immune evasion, is crucial to making clinical decisions aimed at preventing metastasis and enhancing anti-cancer immune responses.

It is likely in early-stage cancers, TDLNs play a crucial role in the generation of anti-cancer immune responses and the prevention of cancer spreading. The presence of TDLNs can create a favorable solution for neo-adjuvant and adjuvant therapy following surgical intervention. However, in late-stage cancers, the infiltration of cancer cells results in an immunosuppressive microenvironment within LNs, which will favor immune evasion and secondary metastases. Of note, patients with advanced cancers are likely to carry distal metastases, which are the primary cause of cancer-related mortality. In such cases, the treatment strategies are likely to target systemic metastases, while the removal of metastatic LNs and targeting of non-metastatic draining LNs may potentially augment the effectiveness of immune checkpoint blockade (ICB) treatment (52, 63). In this review, we discuss the roles of TDLNs in cancer metastasis, the impact of cancer cell progression and invasion on the LN immune microenvironment and the function of TDLNs in immunotherapy, as well as the opportunities and challenges in targeting LN metastasis for improving patient care.

## Cancer metastasis in the lymphatic system

### Lymphangiogenesis in cancer

**Figure 1:**
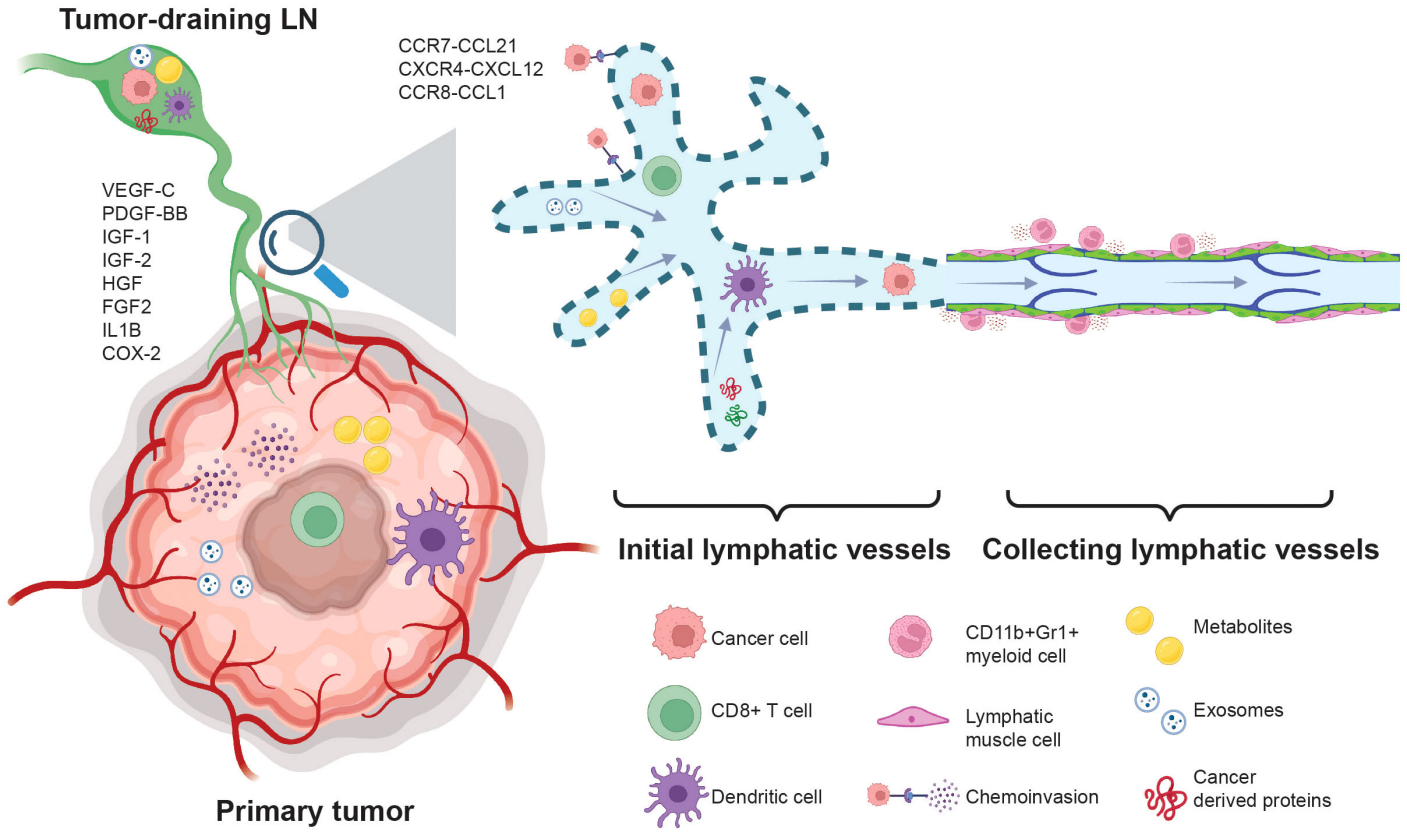
Cancer cell metastasis through the lymphatic system.During cancer progression, cancer-derived cytokines such as VEGF-C, PDGF-BB, IGF1/2, HGF, FGF2, Interleukin 1 (IL1B), and COX-2 promote lymphangiogenesis. Subsequently, lymphangiogenesis accelerates the egress of T cells and suppresses T cell activation via LEC cross-presentation and tolerance. The elevation of VEGF-C drives the expression of CCL21 in LECs, enhancing the chemoinvasion of cancer cells into the lymphatic vasculature. Additionally, chemotactic signals such as CXCR4-CXCL12 and CCR8-CCL1 promote lymphatic metastasis. Lymphangiogenesis increases the transport of cancer cell-derived proteins, exosomes, and metabolites to the TDLN, creating pre-metastatic niches that favor cancer cell invasion. The presence of CD11b+Gr1+ myeloid cells in the tumor-draining collecting lymphatic vessels suppresses lymphatic contraction and reduces tumor-antigen transportation to the lymph nodes.

Tumor growth stimulates both angiogenesis (64) and lymphangiogenesis (65–70), facilitating cancer metastasis through the blood circulation and lymphatic vasculature. Tumor lymphangiogenesis is a prognostic indicator for increased risk of LN metastasis in various cancers, including melanoma (71), head and neck cancer (72–74), and breast cancer (66). Cancer cells and inflammatory cells in the tumor microenvironment secrete lymphangiogenic factors, such as VEGF-C (65, 66), VEGF-D (67), PDGF-BB (69), IGF-1 and IGF-2 (75), HGF (76, 77), FGF-2 (78), interleukin-1 (79), and COX-2 (80), promoting the proliferation and dilatation of peritumoral lymphatics, thereby favoring their metastatic spread (**Figure 1**). In several types of tumors, including fibrosarcoma and melanoma, lymphangiogenesis occurs mainly in the tumor margin. Inside tumors, lymphatic vessels are compressed (68, 81), possibly due to the solid stress exerted by growing cancer cells (32, 44, 45, 55, 56, 68, 82–86), but functional lymphatic vessels in the tumor periphery remain and can transport antigen and metastatic cancer cells. The presence of peritumoral lymphatic vessels accelerates LN metastasis in patients with head and neck cancer by accelerating lymphatic intravasation (72) and actively promotes cancer cell transport to LNs in preclinical models via chemoinvasion that utilizes chemokine signaling pathway such as CCR7 – CCL21 (87, 88), CXCR4 – CXCL12 (89), and CCL1-CCR8 (90) (**Figure 1**).

Lymphatic vessels also play a critical role in transporting antigens and antigen-presenting cells to LNs to initiate an adaptive immune response. Lymphatic endothelial cells can also archive and present antigens (91, 92). However, instead of enhancing tumor antigen transportation and immune cell trafficking to the TDLNs, tumor-associated lymphatic endothelium can impede anti-cancer T-cell response and promote cancer cell immune evasion and metastasis (93–97) (**Figure 1**). In the tumor microenvironment, infiltrating CD8+ T cells produce IFN-γ which drives the expression of PD-L1 on lymphatic endothelial cells (LECs) (94). The presence of PD-L1 on LECs induces T cell tolerance through PD1/PD-L1 interaction and lack of co-stimulation (98), thus restricting CD8+ T cell activation and reducing their accumulation in the tumor microenvironment (95). Additionally, PD-L1 on LECs in the LN prohibited LEC proliferation, protected them from apoptosis, and regulated LN expansion and contraction during inflammation (99). In a murine model of B16F10 melanoma expressing both VEGF-C and chicken ovalbumin (OVA), overexpression of VEGF-C in cancer cells promotes lymphangiogenesis in both primary tumors and TDLNs, leads to CD8+ T cell immune tolerance via LEC cross-presentation (**Figure 1**), protects tumors against preexisting anti-cancer immunity, and promotes the local elimination of OVA-specific CD8+ T cells (93). Mice lacking dermal lymphatic vessels (K14-VEGFR3-Ig mice) with B16F10 intradermal tumors have reduced spontaneous lung metastasis and enhanced tumor infiltration of cytotoxic CD8 T cells (96). The presence of tumor-associated lymphatic vessels also promotes the exit of CD8+ T cells via the CXCL12 – CXCR4 chemotaxis, reducing the retention of tumor-specific CD8+ T cells (97). Pharmacologically blocking CXCR4 or genetically depleting CXCL12 in LECs enhances CD8+ T cell retention and promotes anti-cancer immunity (97).

While lymphangiogenesis is associated with cancer metastasis and poor prognosis, it also improves the therapeutic benefit of immune checkpoint inhibitors. In both murine melanoma models and patients with melanoma, the presence of tumor lymphatic vessels showed a strong correlation with a local inflammatory response (96). Despite the immunosuppressive microenvironment of lymphangiogenic tumors mediated in part by LEC antigen presentation-induced T cell tolerance (94, 95, 98), they are more sensitive to systemic immunotherapy due to the elevation of CCL21 in LECs, which promotes the retention of naïve T cells and DCs in lymphangiogenic tumors via CCL21/CCR7 chemokine axis (100). Of note, in the B16F10 melanoma model, a high density of effector T cells in the tumor promoted LEC apoptosis via the IFN-γ signaling pathway (92). Subsequently, the decline of tumor lymphatic vessel density restricted lymphatic drainage (92), which might prevent the egress of tumor-specific CD8+ T cells (97). Taken together, cancer-associated lymphatic vessels can potentially drive cancer progression, but also be utilized to improve anti-cancer immunotherapies.

### The impact of cancer cell migration on the lymphatic system

The lymphatic vascular system is a low-pressure, unidirectional circulation system that plays a critical role in tissue fluid homeostasis, tissue regeneration, lipid absorption and transportation, as well as immune surveillance (43, 101). Lymphatic vessels can be divided into initial (capillaries), pre-collecting, and collecting lymphatic vessels (43, 85, 102). The tissue interstitial fluid and macromolecules enter the lymphatic system through initial lymphatic vessels to form lymph and subsequently drain to the collecting lymphatic vessels and LNs. Lymph eventually transports back through the thoracic duct to re-enter the blood (43).

The initial lymphatic vessels are composed of a single thin layer of LECs, forming an oakleaf-like shape with button-like junctions between them (103). The low-pressure characteristic of lymphatic vasculature, along with the button-like junctions of the initial LECs, plays an important role in antigen transportation and dendritic cell (DC) trafficking (104). The initial LECs constitutively express CCL21 to actively recruit DCs via CCL21-CCR7 chemotaxis (105). However, the button-like junction and the presence of CCL21 in LECs also open a path for cancer cells to enter lymphatic vessels (87, 88). Additionally, the initial lymphatic vessels permit the delivery of cancer-derived cytokines (106), shed tumor-derived factors (107), metabolites (108), and exosomes (109–111) to LNs. Consequently, these processes remodel non-hematopoietic cells in the LN (112–114), creating a pre-metastatic lymphovascular niche in the LNs to facilitate cancer invasion.

In the collecting lymphatic vessels, the LECs are interconnected by tight zipper-like junctions and are surrounded by lymphatic muscle cells (103, 115). In addition, the collecting lymphatic vessels also contain intraluminal valves to prevent backflow (116), thus allowing the unidirectional propulsion of lymph. The collecting lymphatic vessels exhibit autonomous tonic and phasic contraction, driving the lymph flow. However, during cancer progression, the accumulation of inducible nitric oxide synthase positive (iNOS+) and CD11b+Gr1+ myeloid cells in the peritumoral region suppresses the contraction of tumor-draining collecting lymphatic vessels (117) (**Figure 1**), which might diminish tumor antigen transportation and DC trafficking to the TDLNs, reducing the immune response (118). The reduction of tumor-draining lymphatic vessel contraction could lead to the elevation of interstitial fluid pressure in primary tumor sites (119, 120), which might drive cancer cells to migrate toward functional lymphatic vessels that are mainly positioned in the tumor margin (68). Of note, *in vitro* experiments demonstrated that the increased pressure can trigger cancer cells to create autocrine gradients of CCR7 ligands and secrete them into the extracellular matrix (ECM), subsequently driving cancer LN metastasis via CCR7-dependent chemotaxis in the direction of flow toward functional lymphatic vessels (121). The extent to which tumor-derived factors can directly suppress lymphatic muscle cell contraction requires further investigation.

### Stromal adaptations in tumor-draining LNs

**Figure 2:**
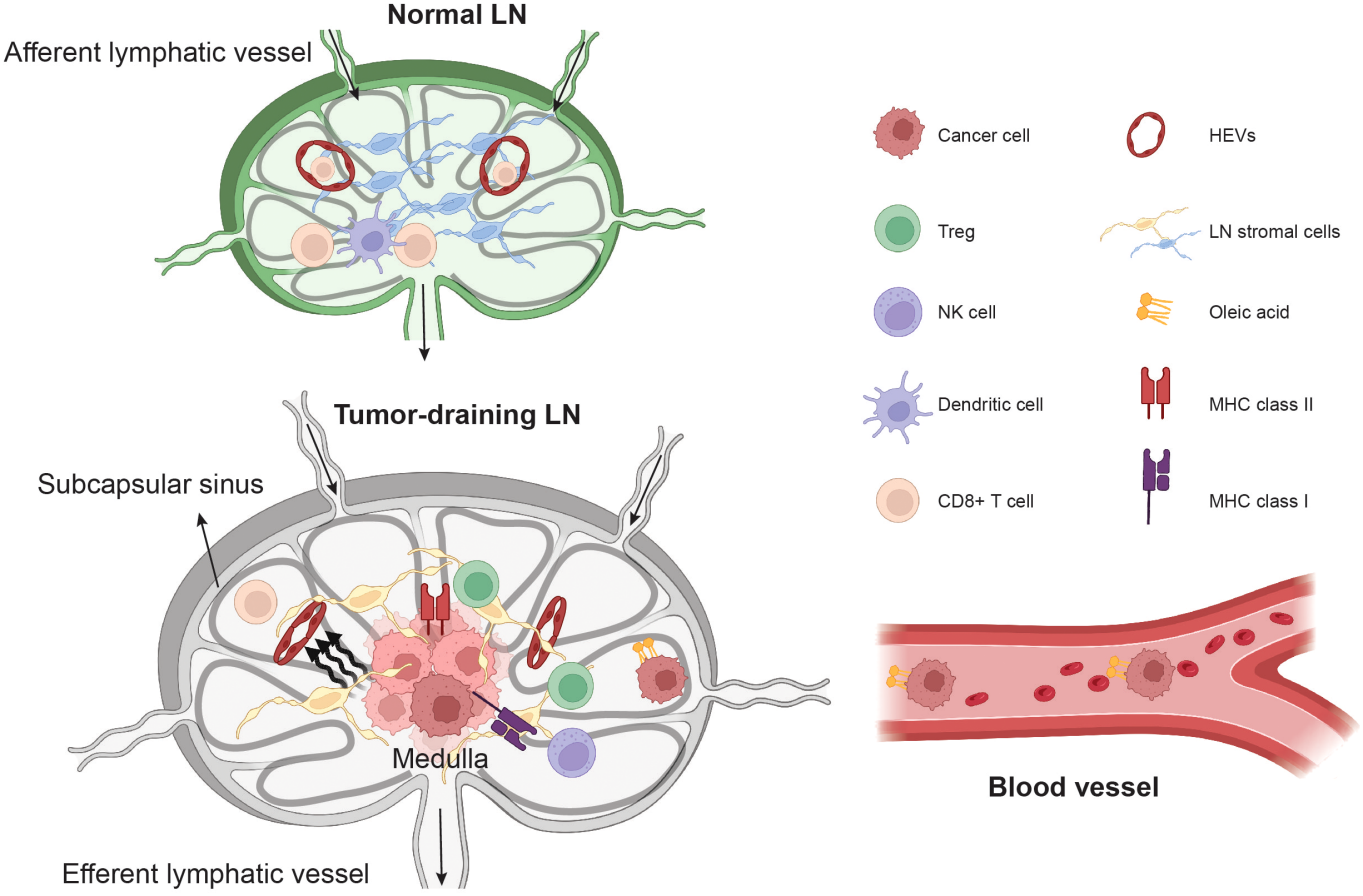
The remodeling of TDLN microenvironment and cancer cells.The invasion of cancer cells leads to the remodeling of stromal cells in the lymph nodes, and cancer cell growth imposes solid stress, which compresses high endothelial venules (HEVs), preventing T cell ingress. In the lymph nodes, cancer cells also undergo metabolic reprogramming, such as the elevation of oleic acid, to gain resistance against ferroptosis in the bloodstream. The unique microenvironment in the lymph nodes drives cancer cell-intrinsic interferon signaling pathways, causing the elevation of MHC class I and MHC class II in cancer cells. This leads to resistance to NK cell killing and the accumulation of Treg cells in TDLNs through anergic mechanisms.

The LN stromal cells provide a framework structure that maintains the niches for immune cells, acts as a backbone for immune cell migration, and forms a conduit system to filter antigens in the lymph (122–125). In addition, the LN stromal cells also provide survival signals and soluble factors that can nourish immune cells to generate strong immune responses (126, 127). The LN stromal cells can be subdivided into FRCs, LECs, blood endothelial cells (BECs), and double-negative (DN) cells (126), based on their anatomical location and gene expression. Furthermore, some blood vessels in the lymph node are morphologically and functionally specialized vessels with thick endothelial cells called high endothelial venules (HEVs) (128). HEVs express high levels of addressins, such as PNAd and MAdCAM-1, which bind L-Selectin/CD62L on lymphocytes and support the migration of naïve lymphocytes from the bloodstream into lymph nodes (128, 129). The FRCs are mainly located in the T cell and B cell zones, with high expression of podoplanin (PDPN) but diminished or absent CD31 endothelial cell marker gene expression (122). The LECs are positioned in the subcapsular sinus, the interfollicular ridges and the medullary sinus (114, 123, 125, 130), expressing both PDPN and CD31. The BECs are positive for CD31 but lack PDPN gene expression (131). Advancements in single-cell RNA sequencing have characterized previously unrecognized spatial heterogeneity and immune functions of stromal cells in the lymph nodes (114, 122, 123, 125, 130, 131), suggesting that the disintegration of the highly organized stromal cell structure will impair the anti-cancer immune responses (132). We have shown that the growth of metastatic breast cancer cells in LNs imposes solid stress on HEVs and prevents the infiltration of T cells into the metastatic lesions (56) (**Figure 2**). Moreover, in both 4T1 breast cancer and B16F10 melanoma models, LN LECs undergo structure and gene expression remodeling even before cancer cell invasion (113). Tumor invasion causes the expansion of the FRC network and the down-regulation of CCL21 and IL-7, leading to immunosuppressive features (112). In human lymphoma, the transcriptomic remodeling of non-hematopoietic cells has been reported, including the upregulation of CD70 in the medulla stromal cells which drives the accumulation of CD27-positive malignant B cells in the medullary regions (114). Taken together, stromal cell remodeling in LNs is one of the key events that precedes and occurs during LN metastasis and lymphoma progression (**Figure 2**). Uncovering the heterogeneity and plasticity of stromal cells in both immune-activated and immune-suppressed lymph nodes offers potential diagnostic markers to complement lymph node biopsy in clinical settings.

### Metabolic adaptations during lymphatic metastasis

Metabolic reprogramming supports the diverse energetic and proliferative demands of cancer cells but also creates unique metabolic shifts that can influence disease progression and therapeutic targets. Tumors exhibit heterogeneous metabolic functions driven by a combination of cell-intrinsic factors and extrinsic influences from the tumor microenvironment (133–135). Metabolic and lipidomic processes significantly influence cancer cell progression and metastasis (136). For example, metabolic pathway alterations in cancer cells, such as increased lactate uptake and amino acid metabolism shifts, support metastasis and cancer progression (137, 138). Moreover, cancer cells upregulate antioxidant pathways to maintain redox balance amidst oxidative stress and mitochondrial dysfunction, crucial for their survival (139–141). Metabolism also plays a critical role in altering immune cell responses, thus exacerbating cancer progression (134, 142). Cancer cells can secrete metabolites and lipids that alter the function of surrounding fibroblasts and compromise immune cell function (143). For example, lipid droplet accumulation in cancer cells has been shown to inactivate immune signaling molecules and foster a hospitable environment for cancer cell progression (144). Additionally, lipids such as prostaglandins can modulate immune responses and promote angiogenesis, further supporting tumor growth and metastasis (145).

Although metabolomic and lipidomic techniques are now being applied to understand the role of metabolites and lipids in the lymph node microenvironment related to cancer metastasis, much remains to be explored. Innovations in these technologies have enabled profiling from low cell number samples (146, 147), allowing for the study of both cancer cell and immune cell populations from metastatic sites including TDLNs. Recent work utilizing these low cell number metabolomic and lipidomic techniques has shown that the distinct microenvironment of the lymph induces metabolic and lipidomic alterations of cancer cells which enhances the establishment of secondary metastases (62). The lymph fluid of mice has higher levels of glutathione and oleic acid, and less free iron compared to plasma. These conditions reduce cancer cell lipid oxidation and promote resistance to ferroptosis in lymph node metastases in both immunocompromised mice with humanized melanomas and immunocompetent mice with melanomas. Metabolomic analysis revealed that melanoma cells in the lymph compared to blood exhibited increased lipid metabolism; in particular, melanoma cells in lymph had higher oleic acid content in membrane phospholipids. The acyl-CoA synthetase long-chain family member 3 (ACSL3), which is critical in converting fatty acids into fatty acyl-CoA esters, enables cancer cells in the lymph to incorporate oleic acid in the cell membrane to protect them from ferroptosis (62).

Within the context of lipidomic dependencies in lymph nodes, recent studies have shown that a subpopulation of pre-metastatic oral carcinoma cells expresses high levels of the fatty acid receptor CD36 and lipid metabolism genes (148). These cells rely on dietary lipids to promote metastasis, and blocking CD36 significantly inhibited lymph node metastasis, underscoring the critical role of lipid metabolism in the metastatic process (148). Further comparative transcriptomic and metabolomic analyses of primary and LN-metastatic tumors in mice with melanoma found that LN metastases require a metabolic shift toward fatty acid oxidation (FAO) (61). Yes-associated protein (YAP) is selectively activated in metastatic lesions in LNs, which leads to increased transcription of genes in the FAO signaling pathway. Genetic deletion of YAP or pharmacological inhibition of FAO suppressed LN metastasis in mice (61). Furthermore, tumor-derived lactic acid alters the metabolic status of TDLN stroma to facilitate metastasis (108). These studies show that metastatic cancer cells in lymph nodes compared to cancer cells from the primary tumor undergo metabolomic and lipidomic alterations that confer survival advantages for metastasis. Considering that current cancer diagnoses primarily rely on features observed in cancer cells at primary sites, gaining a deeper understanding of the lipidomic and metabolic adaptations that occur during LN metastasis could offer novel solutions to prevent the spread of cancer cells. The advancements in single-cell transcriptomics and spatial metabolomics provide novel solutions to address the metabolic evolution of cancer and immune cells during LN metastasis.

### LN metastasis and systemic metastasis

Different models and mechanisms of cancer metastasis have influenced clinical practice, including the role of LN metastases as predictors of distant metastasis. LN involvement has been historically considered a crucial step that preceded further metastasis, but contemporary insights suggest that distant metastases may not solely originate from LN-seeded cells. In the prevalent sequential progression model, cancer cells first spread to regional LNs and then disseminate to distant organs via the lymphatic system (149). In this model, LN metastasis serves as an important prognostic factor and triggers lymphadenectomy to prevent tumor cells from spreading further to distant organs (16, 19, 21, 150–152). However, in colorectal cancer (153–155) and breast cancer (156–158), evolutionary analysis of synchronous LN metastases and distant metastases indicates that only a small portion of patients share clonal ancestry between their LN metastases and distant metastases. In colorectal cancer, ~65% of lymphatic and distant metastases arose from independent subclones in the primary tumors, whereas ~35% of cases shared a common subclone origin (153, 155). Evolutionary analysis of matched breast cancer primary and metastatic tumors from 10 patients demonstrates the presence of both monoclonal and polyclonal origins of metastasis (156). Taken together, these data suggest that both direct hematogenous metastasis from the primary tumor and sequential progression from LNs can occur. Being able to determine which processes are occurring in an individual patient is a current unmet challenge.

Evolutionary studies of cancer in solid tumors reveal that LN metastases display higher levels of genetic diversity than distant metastases (154, 159), suggestive of their polyclonal nature (160). In colorectal cancer, LN metastases exhibit higher levels of inter-lesion and intra-lesion diversity than distant metastases (154). Additionally, colorectal cancer LN metastases develop through a wide evolutionary bottleneck, resulting in polyclonal and polyphyletic phenotypes. Distant metastases form monophyletic groups and have reduced clonality compared to LN metastases, suggesting different evolutionary mechanisms between LN metastases and distant metastases (154). A similar phenomenon was observed using a molecular barcoding system in a murine breast cancer spontaneous metastasis model, which showed LN metastases have higher levels of clonal diversity than distant metastases (159). These evolutionary studies reveal that fewer clones of cancer cells from the primary tumor site can form metastases in distant organs compared to TDLNs, which might be attributed to the different selective pressures and microenvironments in these organs, such as the varying metabolites in distant organs (161, 162). Meanwhile, cancer cells need to travel a longer distance to establish metastasis in distant organs than in locoregional lymph nodes. Since, both human (153, 155) and murine (163, 164) LN metastases can seed distant metastases, the abundance of heterogeneous cancer cell subclones in LNs could potentially give rise to more treatment-resistant cancer cells, such as the ferroptosis-resistant clones (62) or immune-resistant clones (57, 59), which spread systemically and generate secondary metastases. Further study is needed to understand the consequences of the greater diversity of LN metastases, as well as the selective pressures in different organs.

In the early stage of cancer progression, the sentinel LNs likely retain anti-cancer immunity to prevent cancer cell intravasation and metastasis through blood circulation. This hypothesis is supported by the fact that both breast cancer (165) and melanoma (166) patients with micrometastases (<= 0.1mm in melanoma, and <=0.2mm in breast cancer) showed the same clinical outcomes as patients with a negative sentinel LN biopsy. However, in later stage cancer, TDLNs are largely immune suppressed by soluble factors and exosomes from the primary tumor and metastatic cancer cells (53–55, 59, 167–169). The suppression of TDLNs leads to the induction of tumor antigen-specific Treg cells and the reprogramming of leukocytes. Adoptive transfer of leukocytes from metastatic lymph nodes into tumor-naïve recipients that were then challenged with a model of experimental metastasis showed enhanced progression of metastases in the lung, suggesting systemic immunosuppression caused by leukocytes from the metastatic lymph nodes (57). Thus, the strong predictive ability of established LN metastases may be due to the immune suppression induced by these lesions.

## TDLNs in immunotherapy

### TDLNs in anti-cancer immune response

**Figure 3:**
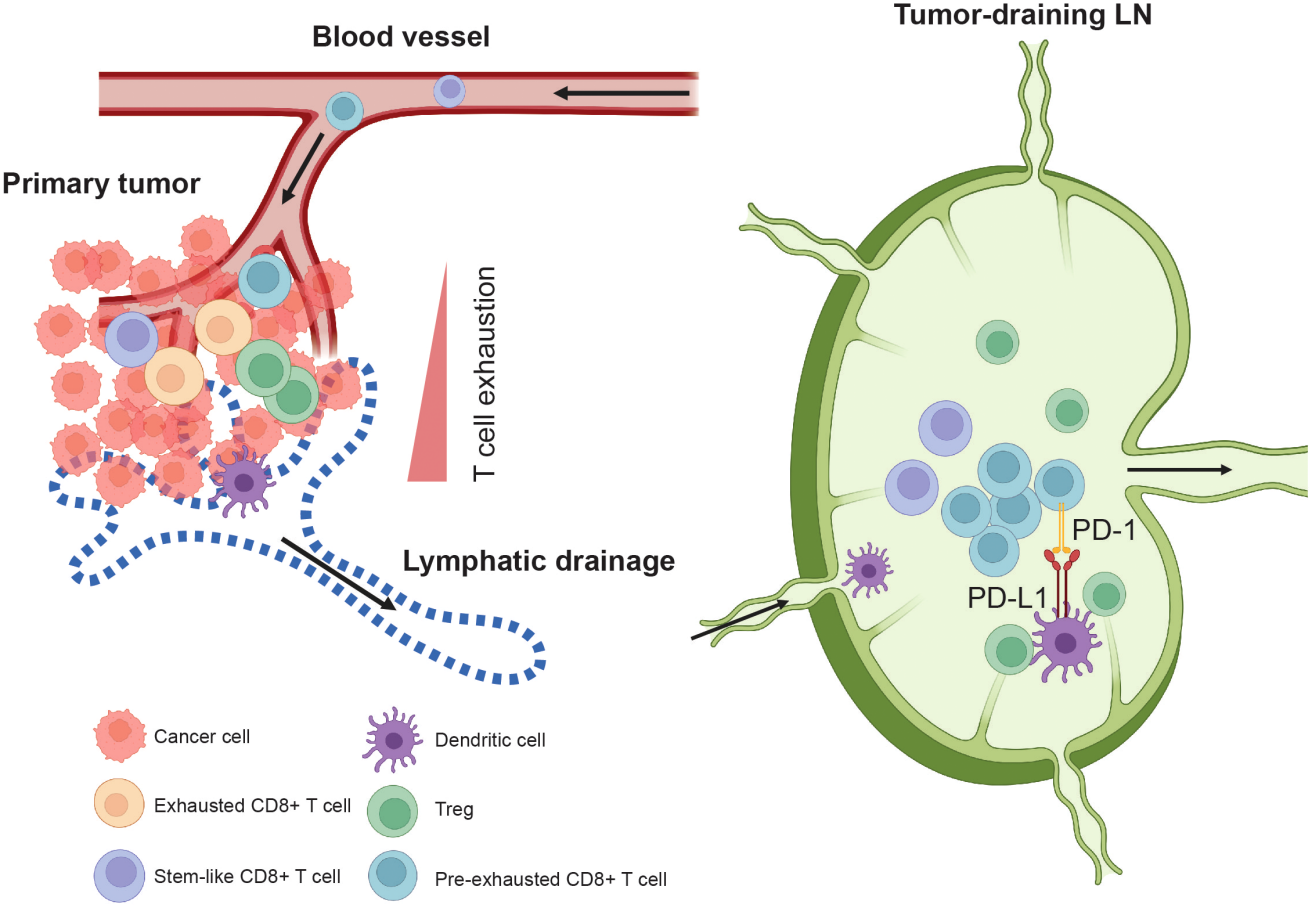
Tumor-draining lymph node in anti-tumor immune responses. TDLNs are the major site where dendritic cells (DCs) present tumor antigens to T cells. Pre-exhausted CD8+ T cells (Tpex) are enriched in the TDLNs and expand after immune checkpoint blockade (ICB) treatment. For example, in the TDLNs, tumor-specific PD-1+ T cells interact with PD-L1+ DCs. Administering PD-L1 inhibitors to TDLNs enhances anti-tumor T cell immunity and accelerates the infiltration of Tpex CD8+ T cells into the tumor, leading to improved tumor control. The stem-like TCF1+ CD8+ T cells do not form a stable population in the tumor. Instead, they continuously egress to the TDLNs via lymphatic drainage. From the TDLNs, they travel through efferent lymphatics, enter the blood circulation, and then circulate back to the tumor, replenishing the population within the tumor.

In the last decade, the landscape of cancer treatment has undergone significant changes, primarily driven by the progress of immunotherapy. However, despite these advancements, a significant challenge that persists is the subset of patients who are resistant to or have limited response to immunotherapy (170), particularly in patients with low tumor mutational burden and immunogenic neoantigens (171, 172). Further, studies show that about 20 ~ 30% of patients with metastatic melanoma initially respond to immune checkpoint blockade treatment but eventually develop resistance and relapse after therapy (173, 174). The acquired resistance to ICB treatment might be due to cancer cell-intrinsic and -extrinsic changes, including the loss of MHC class I on cancer cells (175, 176), genetic mutations that diminish T-cell infiltration (177, 178), or host microbiota (179–182). The underlying mechanisms responsible for the lack of response among patients are yet to be fully understood. Many researchers have focused on investigating the impact of ICB treatment on the interactions between immune cells and cancer cells in the tumor microenvironment (170, 183–185), including the tertiary lymphoid structures (186–190), as well as the T cell phenotypes in the peripheral circulation (48, 50, 191–197). Recently, TDLNs have received more attention due to their normal function as an immune organ. Several studies demonstrated that TDLNs play a critical role in anti-cancer immunity upon ICB treatment (30, 31, 33, 34, 36, 47, 51, 198, 199) (**Figure 3**). Traditionally, lymph node biopsy is primarily employed for cancer staging and serves as a prognostic marker in the clinic. These new studies suggest that examining the immune microenvironment phenotypes of lymph nodes might be useful in predicting the efficacy of ICB treatment.

One key determinant of ICB treatment efficacy is the quantity and quality of infiltrating CD8+ T cells identified before or during the early stages of treatment (183). After ICB treatment, newly generated CD8+ T cells that infiltrate the tumors displayed different TCR clones compared to the pre-treatment intratumoral CD8+ T cells (50, 197, 200, 201). T cell exhaustion is a state of dysfunction that occurs during chronic infection and cancer. It is characterized by reduced effector function, the expression of inhibitory receptors, and specific transcriptional pathways that differ from those in effector or memory T cells (202). These ICB pre-treatment CD8+ T cells in the tumors exhibit reduced effector function and lack of proliferation after ICB treatment, indicating that the cells are exhausted (201). In a clinical trial (NCT02919683) of patients with head and neck squamous cell carcinomas of the oral cavity, neoadjuvant anti-PD1 or anti-PD-1/anti-CTLA4 treatment promoted the expansion of anti-cancer CD8+ T cell clones that did not exist in the tumors before the treatment (197), suggesting that these newly generated anti-cancer CD8+ T cells are derived from the TDLNs and not from the expansion of pre-existing exhausted CD8+ T cells in the tumor. Using Kaede photoconvertible mice, experiments demonstrated that CD8+ effector T cells that enter and stay in the tumor develop an exhausted phenotype within 72 hours, whereas non-effector TCF1+ stem-like CD8+ T cells are continuously trafficked between the tumor and the TDLNs (47). In brief, the stem-like TCF1+ CD8+ T cells do not form a stable population in the tumor; they continuously egress to the TDLNs via lymphatic drainage (47, 97) (**Figure 3**). From the TDLNs, these stem-like TCF1+ CD8+ T cells travel through efferent lymphatics and then enter the blood circulation and circulate back to the tumor to replenish this population (47) (**Figure 3**). Analysis of CD8+ T cells from primary human head and neck squamous cell carcinomas, regional LNs and blood shows that the non-metastatic TDLNs are the source of pre-exhausted CD8+ T cells (Tpex), which migrate to the tumor (52). Similar findings were discovered in preclinical animal models of lung cancer (31) and melanoma (199), in which most tumor-specific CD8+ T cells in the TDLNs are functional and exhibit a gene expression signature resembling that of stem-like CD8+ T cells (31) or resident memory CD8+ T cells (199), suggesting that the antigen presentation and T cell priming in TDLNs is critical for the ICB treatment.

PD-1/PD-L1 checkpoint-blocking antibodies are thought to act primarily in the tumor microenvironment, where PD-L1 is expressed by tumor cells (174), myeloid cells (168, 203, 204), LECs (94, 95, 98), blood endothelial cells (95), B cells (205, 206), T cells (207), dendritic cells (30, 203) and NK cells (208, 209). An evaluation of the role of TDLN in PD-1/PD-L1 checkpoint blockade therapy in two mouse tumor models reveals that immune checkpoint treatment induces CD8+ T cell accumulation in the tumor-draining but not in non-draining LNs (34). Surgical removal of these TDLNs abolished PD-1/PD-L1 checkpoint blockade-induced tumor regression and was associated with decreased immune infiltration in the primary tumors (34). Moreover, the immunomodulator FTY720—which blocks the egress of lymphocytes from secondary lymphoid organs and thymus by agnostic activation of sphingosine 1 phosphate receptors (210)—abrogated checkpoint therapy, suggesting that TDLNs function as sites of T cell invigoration required for PD-1/PD-L1 checkpoint blockade therapy (34). PD-L1 can interact with other binding partners such as CD80 to form heterodimers on the same cell (211, 212). In APCs, CD80 interacts with PD-L1 *in cis* to disrupt PD-L1/PD-1 interaction between APCs and T cells, limiting the PD-1 coinhibitory signal, while promoting CD28/CD80 interactions and enhancing T cell activation (213). Meanwhile, the presence of PD-L1 on T cells may also interact with CD80 on APCs (214) leading to tumor-promoting tolerance through varying mechanisms (207). Notably, TDLNs are enriched for tumor-specific PD-1+ T cells, which frequently interact with PD-L1+ conventional dendritic cells (30). Targeting TDLNs with PD-L1 inhibitors induces enhanced anti-cancer T cell immunity and accelerates the infiltration of progenitor-exhausted T cells into the tumor, leading to improved tumor control in mice (30) (**Figure 3**). Given that a variety of cells in the lymph nodes possess the ability to express PD-L1, including LECs (94, 95, 98), FRCs (215), dendritic cells (30, 203), macrophages, B cells, T cells, and NK cells (204, 208, 209), understanding the impact of PD-1/PD-L1 immune checkpoint blockade on these cells and their interactions with T cells in the TDLNs might provide novel solutions to further enhance the efficacy of immunotherapy. In summary, TDLNs are crucial for the maintenance and trafficking of tumor antigen-specific Tpex and stem-like CD8+ T cells. These cells can be primed and activated after ICB treatment, enabling them to migrate to the tumor site and exert their effector function on cancer cells.

### Immune evasion in the LNs

Despite TDLNs being recognized as key organs in response to immunotherapy and modulating anti-cancer immunity, emerging data suggest that TDLNs are often immunosuppressed by cancer cells. In melanoma, cancer cells inhibit the activation of different DC subsets (169), including the CD5+ DC (58), and suppress immune activation in the LNs. The accumulation of Treg cells in TDLNs has been reported in several different types of solid tumors, including breast cancer (55, 59, 216), lung cancer (53), colorectal cancer (167), melanoma (57, 169), gastric cancer (217), head and neck cancer (218), and cervical cancer (168). Recently, we showed that the invasion of breast cancer cells in the LN blunts the CD4+ effector T cell proliferation and promotes the expansion of Treg cells (59). Mechanistically, we uncovered that breast cancer cells in the LN displayed high levels of interferon-induced gene signatures, including a subpopulation of cancer cells that had MHC-II gene expression and an absence of co-stimulatory molecules (**Figure 2**). Of note, the presence of MHC-II in cancer cells is not restricted to breast cancer. It has also been reported in other types of solid tumors (219), including colorectal cancer (220), lung cancer (221, 222), ovarian cancer (223) and melanoma (224, 225). Additionally, MHC-II is present in LECs within tumors and lymph nodes (43, 226–228), as well as the cancer-associated fibroblasts in pancreatic cancer (229). Without co-stimulatory signals on the antigen-presenting cell (in this case the cancer cell), CD4+ T cells will not differentiate into effector cells in response to antigens, which prevents an effective immune response. Subsequently, we reported that the MHC-II+ cancer cells suppress Th1 responses and promote the expansion of Tregs in the LNs. Furthermore, we found that depletion of MHC-II in 4T1 breast cancer cells significantly reduced LN metastasis and attenuated Treg cell expansion in the TDLNs. In contrast, overexpression of *Ciita*—a transcriptional activator of MHC-II—in 4T1 breast cancer cells promoted LN metastasis and enhanced Treg cell expansion in the TDLNs (59). While not shown, it is possible that the Treg cells in metastatic LNs subsequently enter the bloodstream to suppress systemic anti-cancer immunity and promote the establishment of distant metastases (230).

Tregs can also directly inhibit natural killer (NK) cell effector functions by the down-regulation of NKG2D receptors on the surface of NK cells caused by membrane-bound TGF-β on Tregs (231). NKG2D receptor downregulation impairs the ability of NK cells to recognize tumor cells. The depletion of mouse Treg cells enhanced NK cell proliferation and cytotoxicity *in vivo* (231, 232). Depletion of Treg cells from tumor-infiltrating lymphocytes also restored human NK cell-mediated tumor recognition (231). In a syngeneic melanoma mouse model featuring a serial selection of cancer cells with a preference for dissemination to the LNs, Reticker-Flynn et al. revealed that the LN microenvironment can reprogram cancer cells, leading to an elevation in interferon-induced gene signatures, which subsequently caused the upregulation of MHC class I and PD-L1 gene expression in cancer cells (57). The elevation of PD-L1 and MHC-I shielded melanoma cells from attack by (NK cells). They also found more Treg cells in LNs invaded by melanoma cells **Figure 2**. The tumor antigen-specific Treg cells subsequently leave the LN and systemically disseminate, thus rendering distant organs more hospitable to metastatic seeding (57). Similarly, in a murine breast cancer LN metastasis model, the expansion of Treg cells in LNs suppresses NK cell activity leading to breast cancer metastasis in LNs (216). Immune-activated T and B lymphocytes typically migrate to sites of inflammation rather than remaining in LNs for extended periods. Thus, NK cells, not T and B lymphocytes, may also participate in immune responses against targets inside LNs, opening an opportunity to target LN metastasis using NK cells.

## Opportunities and challenges in targeting TDLNs

### LN-targeted immunotherapy

As TDLNs are widely recognized for playing a key role in driving the immunotherapy response, it is hypothesized that the effectiveness of ICB relies predominantly on an influx of newly primed T cells into the tumor from peripheral lymphoid organs, particularly the TDLNs, rather than on reinvigorating exhausted and fully differentiated T cells that already exist in tumors (31, 33, 47, 52). Thus, leveraging the CD8+ T cell pools in the TDLNs is one of the promising approaches in immunotherapy. In the B16F10 melanoma model, directing anti-CTLA4 and anti-PD-1 treatment to the TDLN reduces the risk of immune-related adverse events, reduces distant metastases and recurrence, and enhances the anti-cancer T cell repertoire (233). A phase 1 clinical trial evaluated the efficacy of locally administering anti-CTLA-4 into TDLNs in patients with stage I/II melanoma (NCT04274816) (36). The results showed activation of migratory dendritic cells in sentinel LNs, an increase in effector T cells, and a decrease in Tregs in both sentinel LNs and peripheral blood (36). These findings suggest that local administration of ICB regimens is a safe and promising adjuvant treatment strategy for patients with early-stage melanoma. In preclinical breast cancer models, the CD8+ T cell viability and responses within the primary tumor were severely impaired. However, CD8+ T cell priming within LNs, which is dependent on lymphatic drainage from the primary tumor, remained intact (32). Therefore, TDLNs represent an important component of the cancer immunity cycle in melanoma and triple-negative breast cancer for which strategies may be developed to improve the effects of anti-PD-1 immunotherapy (32) (**Figure 3**). Notably, TDLN-derived lymphocytes with abundant tumor-reactive pre-exhausted T cells (52) are relatively easy to obtain. This has been utilized in several phase I/II trial (NCT05981014, NCT06121570, NCT06121557) that investigate the safety and effectiveness of infusion of autologous lymphocytes derived from TDLNs in patients with HER2-negative breast cancer who did not respond to neoadjuvant chemotherapy.

Chemoimmunotherapy is an experimental therapeutic approach for cancer that combines traditional chemotherapy, such as paclitaxel, with immunotherapy. Mechanistically, this approach leverages chemotherapy to induce immunogenic cell death, whereby the release of danger signals and tumor antigens boost the anti-cancer efficacy of immunotherapy (234). LN-targeting nanotechnology that delivers the chemotherapeutic drug paclitaxel to direct chemoimmunotherapy to TDLNs resulted in T cell mobilization into the circulation and improved control of both primary and metastatic tumors as well as animal survival in multiple murine models of triple-negative breast cancer (35). These data show that TDLNs mediate the effects of chemoimmunotherapy in advanced murine breast cancers (35). Further, a pre-clinical study of metastatic lung cancer reported that TDLNs help control metastases after the primary tumor is removed but are not required for adjuvant immunotherapy efficacy (235). Using orthotopic murine breast cancer and melanoma that develop spontaneous LN metastases, we also showed that resection of TDLNs did not prevent ICB responses (63), which was attributed to the rerouting of antigen drainage to distant LNs. It is worth noting that these pre-clinical studies were conducted in mice, whose tumor lymphatic drainage and lymphosome are distinct from those of humans. The rate of positive SLNB after neoadjuvant ICB therapy in patients with melanoma or breast cancer will be an interesting secondary endpoint to follow.

Tumor-derived secreted factors preferentially drain to lymphatic vessels before dilution in the blood. A recent study examined the cancer-derived secreted factors in lymph exudate in patients with metastatic melanoma after lymphadenectomy. This work demonstrated that tumor-derived extracellular vesicles were enriched in the lymph exudate (110), suggesting that exosomes may offer a novel solution for drug delivery to the LNs. However, tumor-derived exosomes often cause stromal remodeling in the LNs to facilitate lymphatic metastasis by cancer cells (109). To overcome this, a recent strategy involved creating macrophage-tumor hybrid cells by introducing nuclei isolated from tumor cells into activated M1-like macrophages, resulting in the production of chimeric exosomes. These exosomes entered LNs and primed T cell activation through both the classical antigen-presenting cell–induced immunostimulatory pathways and a unique ‘direct exosome interaction’ mechanism (236). Of note, despite the interesting concept presented in this study, the clinical implementation of this strategy remains challenging.

### LN-targeted cancer vaccination

Tumor-specific vaccines are emerging as a promising strategy to prevent tumorigenesis, as well as suppress the growth of existing tumors (39). Delivering these vaccines more efficiently to TDLNs, which are often the first sites where lymphocytes are presented with tumor-associated antigens, is under extensive exploration (38, 40, 41, 237–244). Intranodal administration of antigen plasmids (pMEL-TYR) and peptides (E-MEL and E-TYR) against Melan A Tyrosinase in patients with metastatic melanoma yielded an overall immune response rate of 50% (243). In both the B16F10 melanoma and E.G7-OVA lymphoma model, TDLN-targeting nanoparticle (NP)-conjugate vaccines induced substantially stronger local and systemic cytotoxic CD8+ T-cell responses when compared to non-TDLN-targeting vaccination, leading to enhanced tumor regression and host survival (237). Amphiphile modification improves the delivery of conjugated cancer vaccines and adjuvants to the lymph nodes, leading to 30-fold increases in T-cell priming and enhanced anti-tumor efficacy (238). A recent study demonstrated that amphiphile modification of G12D and G12R mutant KRAS (mKRAS) peptides (Amph-Peptides-2P) in combination with CpG oligonucleotide adjuvant (Amph-CpG-7909) enhanced the delivery of cancer vaccines to lymph nodes and promoted anti-tumor immune responses in pancreatic and colorectal cancer (38). In addition, other strategies have also been developed to enhance the lymph node-targeted vaccination, including the pullulan nanogel system (240), synthetic vaccine nanoparticles (239), LN-targeted lipid nanoparticles (242, 245), and LN-targeted immunization formulation (244, 246, 247). For example, oil immunization led to rapid transportation and retention of antigens in the interfollicular region of the LN, whereas without oil, the antigens were enriched in the medullary region (246). The PEGylated lipid NPs together with mannose facilitated the phagocytosis and antigen presentation by dendritic cells in the lymph nodes after subcutaneous administration (244). PEGylated reduced graphene oxide nanosheets (RGO-PEG, 20–30 nm in diameter) rapidly accumulated in LNs, elicited neoantigen-specific T cells and eradicated established MC-38 tumor (37). Furthermore, in the murine melanoma model, mesoporous silica (MPS) rod-based vaccines enhanced durable expansion of lymph nodes and were associated with better and prolonged vaccine efficacy (247). Beyond LN-targeted cancer vaccines, amphiphile CAR-T ligands delivered to the lymph nodes can be presented by APCs in the native lymph node microenvironment to enhance CAR-T cell expansion (40), promote tumor antigen spreading and elicit endogenous anti-tumor T cells (41, 241).

Radiotherapy triggers abscopal effects to control tumor growth (248). In radioimmunotherapy, the synergy between radiotherapy and ICB treatment acts similar to *in situ* vaccination. Recent studies showed that TDLNs play a critical role in the efficacy of radio-immunotherapy (249–251), particularly in the adjuvant setting (251). In a syngeneic hepatocellular carcinoma mouse model, combining radiation and anti-PD-1 enhanced activated dendritic cells in the TDLNs, accelerated infiltration of activated cytotoxic T cells in both irradiated and non-irradiated tumors, and triggered abscopal effects in the non-irradiated tumors (248). In the B16F10 melanoma model, local tumor irradiation improved distant tumor control and was associated with the expansion of total CD8+ T cells and stem-like CD8+ T cells in the TDLNs (249). Interestingly, the delayed (adjuvant) TDLN irradiation enhanced the efficacy of radioimmunotherapy, unlike the concomitant TDLN irradiation which failed to promote the efficacy of radioimmunotherapy (251). Given that the sentinel lymph nodes or TDLNs are often surgically removed during the processes of cancer diagnosis and treatment, a better understanding of the roles of TDLNs and the tumor-draining lymphosomes in cancer vaccination and radioimmunotherapy is needed to enhance treatment efficacy.

### Target lymphangiogenesis to enhance ICB treatment efficacy

VEGF-C-induced lymphangiogenesis promoted immune tolerance in murine melanoma (93, 94). Mechanistically, LECs associated with lymphangiogenesis in tumors or the draining LN promoted immune tolerance by eliminating anti-cancer CD8+ cells (95) and accelerating T cell egress (97). Thus, LECs in the local tumor microenvironment may be a target for immunomodulation. Further, data showed that pharmacologically blocking VEGFR-3 decreases Treg cell accumulation in melanoma (100). However, despite the immunosuppressive microenvironment in highly lymphangiogenic melanoma, heightened sensitivity to ICB treatment and adoptive T cell transfer therapy was measured. Mechanistically, the VEGF-C signaling pathway induces CCL21 expression in LECs, subsequently enhancing the infiltration of CCR7+ naïve T cells and DCs to the tumors (100). In glioblastoma and melanoma brain metastases, ectopic expression of VEGF-C promotes the drainage of meningeal lymphatics and enhances CD8+ T cell activation in the draining deep cervical LNs. Subsequently, activated CD8+ T cells migrate to and enter the tumor, resulting in better tumor control and long-lasting memory responses with anti-PD1 therapy (252). Taken together, these results demonstrate the multifaceted roles of VEGF-C and LECs in the tumor microenvironment. The presence of high levels of VEGF-C and tumor lymphangiogenesis is associated with immunosuppression but might also indicate better efficacy for ICB treatment. This might be due to the enhancement of T cell infiltration and increased lymphatic drainage, APC trafficking, and antigen transportation to TDLNs.

### Challenges of SLNB and CLND

The sentinel LN biopsy is a standard method for the clinical staging of cancer. The decision to remove additional LNs in patients with a positive SLNB or to forgo completion lymph node dissection depends on various factors, including the type and stage of cancer, and individual patient characteristics. Emerging evidence suggests that CLND does not always provide significant clinical benefits (24, 25, 253). In both melanoma (23, 24, 253, 254) and breast cancer (22, 28, 255, 256), CLND did not show an overall survival benefit compared to SLNB alone in early-stage cancer patients. In addition, in patients with early-stage breast cancer (< 5 cm) and minimal sentinel LN involvement (one or more micrometastases < 2 mm), CLND did not show significant overall survival benefit compared to the no LN dissection group (256). In patients with clinically node-negative primary T1 to T3 breast cancer with at least one positive lymph node identified by SLNB, SLNB-only was not inferior to complete axillary LN dissection for recurrence-free survival with the addition of standard-of-care adjuvant systemic therapy and radiation (28). In patients with small primary breast tumors and negative results on ultrasonography of the axillary lymph nodes, it is safe to skip SLNB (257). Due to the limited benefits of CLND and the significantly higher incidence of lymphedema following the procedure (29), frequent follow-up observations, including the use of serial nodal ultrasound, may be offered to patients with low-risk micrometastatic melanoma (258). Currently, there is no cure and no FDA-approved drug for lymphedema making prevention an important clinical consideration. Lymphedema after cancer treatment causes swelling, discomfort, and limited mobility in the arm, hand, or leg on the affected side. It also increases the risk of infection and interferes with daily activities and quality of life. There is an urgent need to find a less aggressive strategy to target LN metastasis to prevent lymphedema and significantly improve the quality of life for cancer survivors while maintaining excellent cancer control.

## Concluding remarks

It is important to note that the optimal management of LN-positive patients remains an active area of research, and personalized treatment approaches are continually evolving. Clinical trials and further studies are crucial to provide more evidence and guidance on the most effective strategies for addressing LN metastasis while maximizing anti-cancer immune responses. In some cases, sparing the LN and exploring alternative treatment strategies may be a practical option. These strategies include targeted therapies, radiation therapy, or systemic treatments such as chemotherapy, immunotherapy, and cancer vaccines. The decision should be made through a multidisciplinary approach involving input from surgeons, oncologists, and other relevant specialists, taking into account the specific characteristics of the patient’s tumor type and individual factors. Ultimately, the choice between LN removal and alternative treatment strategies should be a data-driven, collaborative decision between the patient and their healthcare team. Further clinical trials exploring therapies that maximize cancer outcomes while minimizing lymphedema risk are critical to informing these decisions.
